# Ultraconserved Elements Mark Genes Involved in Multilayered Regulation and Human Development

**DOI:** 10.3390/ijms27219981

**Published:** 2026-11-07

**Authors:** Larisa Fedorova, Georgiy S. Lebedev, Yuriy L. Orlov, Oleh A. Mulyar, Alexei Fedorov

**Affiliations:** 1 CRI Genetics LLC, Santa Monica, CA 90404, USA; lvfedorova3@gmail.com (L.F.); amulyar@crigenetics.com (O.A.M.); 2 Center of Digital Health and AI in Medicine, I.M. Sechenov First Moscow State Medical University of the Ministry of Health of the Russian Federation ( Sechenov University), 119991 Moscow, Russia; lebedev_g_s@staff.sechenov.ru (G.S.L.); orlov_yu_l@staff.sechenov.ru (Y.L.O.); 3 Peoples’ Friendship University of Russia (RUDN University), 117198 Moscow, Russia

**Keywords:** bioinformatics, computational biology, evolution, DNA conformation, ultra-conserved elements

## Abstract

Ultraconserved elements (UCEs) are genomic sequences exhibiting exceptional evolutionary conservation across vertebrate taxa, yet their biological functions remain poorly understood. Here, we investigated the functional and expression characteristics of human protein-coding genes containing UCEs. Observed UCE distributions across Gene Ontology and manually curated, mutually exclusive functional groups of genes were compared with 1000 randomized UCE datasets that preserved natural UCE clustering. UCEs are preferentially located within genes involved in multilayered regulation, including transcription, chromatin organization, RNA processing, and ubiquitin-mediated protein regulation, whereas genes associated with membrane, extracellular, structural, transport, and metabolic functions are relatively UCE-poor. Within enriched groups, UCE accumulation is concentrated in multifunctional developmental regulators rather than in constitutive cellular machinery. Compared with length-matched UCE-free controls, UCE-rich genes showed higher and broader expression across 68 adult tissues and significantly higher prenatal expression in all seven organs examined. This expression excess was greatest in the developing brain and cerebellum and was most pronounced during early prenatal development. UCE-rich transcription factors are specifically enriched in developmental and chromatin-regulatory programs, including HOX-related embryonic patterning. These findings indicate that UCEs preferentially mark genes involved in several levels of cellular regulation and link ultraconservation to multilayered gene regulation and early human development.

## 1. Introduction

Ultraconserved elements (UCEs) represent one of the most striking examples of evolutionary constraint in vertebrate genomes. Thousands of these genomic sequences have remained nearly unchanged across distantly related vertebrate species over hundreds of millions of years, despite being exposed to ongoing mutational processes [1,2,3]. UCEs are distributed throughout the genome and occur predominantly within intronic and intergenic regions [3]. Remarkably, individual UCEs generally share little sequence similarity with one another and lack a common sequence motif that could readily explain their exceptional evolutionary stability [4].

The biological basis of ultraconservation remains unresolved. Recently, Cummins et al. [3] proposed that interactions with multiple targets and/or regulators could impose constraints on sequence evolution. Various functions have been proposed for UCEs, including roles in transcriptional regulation, developmental enhancers, genome organization, and other regulatory processes [2,5,6,7]. Our previous studies demonstrated that UCEs possess unusual nucleotide and dinucleotide characteristics, particularly enrichment of GpC and depletion of GpG/CpC dinucleotides, and that combinations of such sequence features can distinguish UCEs from ordinary genomic DNA with relatively high accuracy [8,9]. These observations suggested that UCEs may represent structurally and functionally distinctive genomic regions with specific DNA conformation rather than simply extreme examples of conventional sequence conservation.

**Figure 1:**
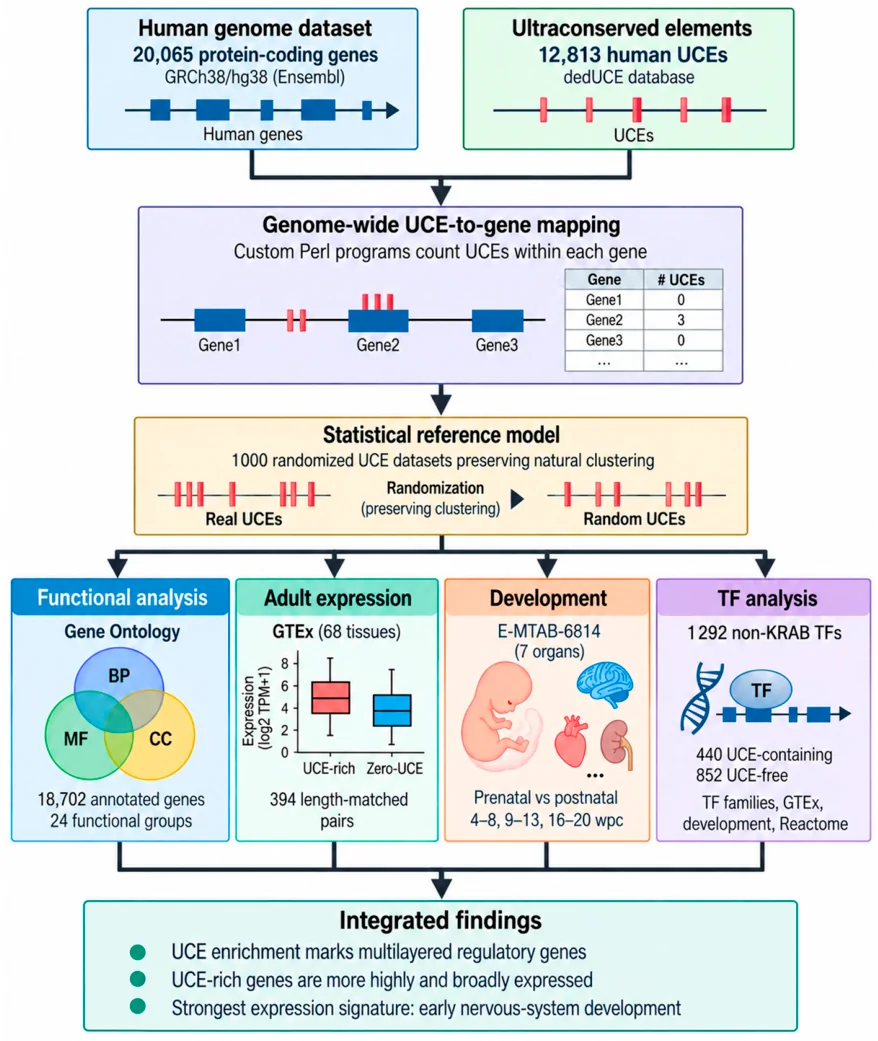
Overview of the computational workflow used for genome-wide analysis of ultraconserved elements in human genes.

More recently, we shifted our attention from the sequence properties of UCEs to their genomic environment. It was demonstrated that UCEs are distributed highly non-randomly among human genes, with particularly strong enrichment in transcription factors and genes involved in developmental regulation [3,10]. These observations raised an important question: what functional and expression characteristics distinguish genes that accumulate UCEs from genes that contain few or no UCEs? In the present study, we address this question through systematic functional classification and analysis of adult and developmental expression patterns of UCE-rich and UCE-free human genes. The workflow of our research is presented in Figure 1.

## 2. Results

### 2.1. Distribution of UCEs Among Genes from Different Functional Groups

We previously demonstrated that UCEs are distributed highly non-randomly within human protein-coding genes [10]. In the present study, we investigate this phenomenon in greater detail across major functional groups of 20,065 human protein-coding genes. UCEs outside these genes were not included in the functional and expression analyses. Gene Ontology (GO) annotations for human genes were obtained and classified into the three major GO domains: Cellular Component, Molecular Function, and Biological Process. These annotations were integrated with our previously generated dataset describing the presence and number of UCEs from the dedUCE database within individual protein-coding genes [10]. This analysis allowed us to evaluate UCE enrichment among genes encoding proteins associated with major cellular components (Table 1), biological processes (Table 2), and molecular functions (Table 3).

To statistically evaluate these distributions, observed UCE counts were compared with expectations obtained from 1000 randomized UCE datasets distributed throughout the human genome while preserving the principal clustering properties of natural UCEs. For each GO category, these simulations provided the expected mean number of UCEs and their standard deviation, which were compared with the corresponding observed values. The observed and expected UCE distributions, together with their statistical parameters, are presented in Table 1, Table 2 and Table 3.

Table 1, Table 2 and Table 3 reveal pronounced differences in UCE enrichment among functional gene categories. The strongest enrichment is consistently associated with nuclear and regulatory functions, including chromatin organization, transcription, RNA processing, and developmental regulation, whereas many membrane-associated, extracellular, transport, and structural categories show relative UCE depletion. However, GO annotations are not mutually exclusive, and the same gene may occur in multiple GO categories. This redundancy can bias interpretation of category-level UCE enrichment. For example, in the GO Biological Process domain there is a group “cellular response to lipopolysaccharide” (Table 3, row 17). It comprises 109 genes harboring 42 UCEs. This group is 2.08 times enriched with UCE compared to random expectation. However, this enrichment is highly concentrated and is predominantly attributable to the *MEF2C* gene, which alone contains 16 of the 42 UCEs in the group. *MEF2C* is also a transcription factor, and its inclusion in the “cellular response to lipopolysaccharide” category reflects one of its broader functional annotations. Removal of *MEF2C* reduces UCE density from 9.16 to 5.91 UCEs/Mb, eliminating the enrichment relative to the genomic protein-coding background. Moreover, most canonical inflammatory cytokines and chemokines (*TNF*, *IL1A*, *IL1B*, *IL6*, *IL10*, *IL18*, *CCL2*, *CCL28*, *CXCL5*, *CXCL8*, *CXCL9*, *CXCL10*, *CXCL13*), inflammasome components, and innate-immune effectors (*NLRP3*, *CASP1*, *PYCARD*, *TICAM1*, *BCL10*, *TNIP1*, *TNIP2*, *TNIP3*) contain no UCEs. Thus, the GO enrichment for cellular response to lipopolysaccharide appears to reflect the presence of a small number of UCE-rich regulatory genes rather than a general association of UCEs with the inflammatory response pathway. This example illustrates why enrichment in overlapping GO categories should be interpreted by examining the contributing genes, rather than treating each enriched term as independent evidence of a broad pathway-level association.

In order to clarify this issue, we created 24 subsets of 7036 human genes with different functionality, which do not intersect with each other and clearly belong to their group. The UCE-richness of these 24 gene groups is presented in Table 4.

The mutually exclusive functional classification in Table 4 independently confirms the broad pattern observed in the GO-based analyses, indicating that the enrichment is not simply a consequence of overlapping GO annotations. UCE enrichment also extends beyond transcription itself: mRNA splicing via the spliceosome is enriched 5.09-fold, heterochromatin formation 4.37-fold, chromatin remodeling 4.11-fold, and regulation of DNA-templated transcription 4.24-fold (see Table 3). In Table 4, the RNA-binding/RNA-processing and chromatin/epigenetic groups show higher UCE enrichment than the combined transcription-factor group (4.70-, 4.01-, and 3.50-fold, respectively). The latter group includes both non-KRAB transcription factors, which showed approximately sevenfold enrichment in our previous study, and UCE-depleted KRAB-ZNF genes (Fedorova et al., 2026) [10].

Conversely, categories involving cell-surface receptors, membrane transport, extracellular interactions, cytoskeletal functions, and metabolic enzymes generally show UCE depletion, although not every individual category reaches statistical significance. Thus, UCEs preferentially occur in genes involved in complex cellular regulatory processes rather than in genes primarily associated with structural, metabolic, membrane-associated, or extracellular functions. The enrichment observed among ubiquitin-related genes is also notable because the ubiquitin system regulates the stability and activity of many transcription factors, chromatin regulators, and developmental proteins. This pattern is therefore consistent with preferential localization of UCEs within genes involved in several levels of cellular regulation rather than with protein degradation per se.

### 2.2. Analysis of UCE-Enriched Gene Groups

Below, we investigate genes from four major UCE-enriched functional groups. Because the number of UCEs is several times smaller than the number of genes, even strongly UCE-enriched groups contain many genes with no UCEs. Thus, only about one-third of genes in UCE-enriched groups harbor ultraconserved sequences, whereas approximately two-thirds are UCE-free. In this section, we focus on genes containing the largest numbers of UCEs and compare them with UCE-free genes from the same functional groups. Because transcription factors constitute the largest and most heterogeneous group, encompassing numerous subclasses, they are considered separately at the end of the Section 2.

#### 2.2.1. UCEs Within Genes Encoding Chromatin and Chromatin-Related Proteins

We examined three groups of chromatin-related proteins: (1) chromatin remodeling from Table 3, with 4.11-fold UCE enrichment; (2) heterochromatin formation from Table 3, with 4.37-fold enrichment; and (3) Chromatin & Epigenetics from Table 4, with 4.03-fold enrichment. After removal of duplicate genes shared among these three overlapping groups, the combined dataset contained 433 unique genes harboring a total of 512 UCEs.

Within the chromatin-remodeling group, UCE enrichment was concentrated in a subset of regulatory genes. The leading genes included *SATB1* (20 UCEs), *NIPBL* (17), *SATB2* (16), *BCOR* (16), *BAZ2B* (12), *CHD7* (10), *KDM6A* (9), *ARID2* (9), and *ARID1A* (8). *SATB1* was particularly UCE-dense, containing 20 UCEs within approximately 100 kb (~200 UCEs/Mb). Many of these genes are involved in developmental transcriptional control and higher-order chromatin organization.

The heterochromatin-formation group contained several prominent UCE-rich genes, including *AUTS2* (42 UCEs), *BCOR* (16), *BAZ2B* (12), *LCOR* (11), and *SUZ12* (6). In contrast, many canonical components of heterochromatin formation and maintenance, including *HDAC1*, *HDAC2*, *KDM2B*, *RBBP4*, *RBBP7*, *RNF2*, *RING1*, *CBX3*, and *PCGF*-family genes, were UCE-free.

**Figure 2:**
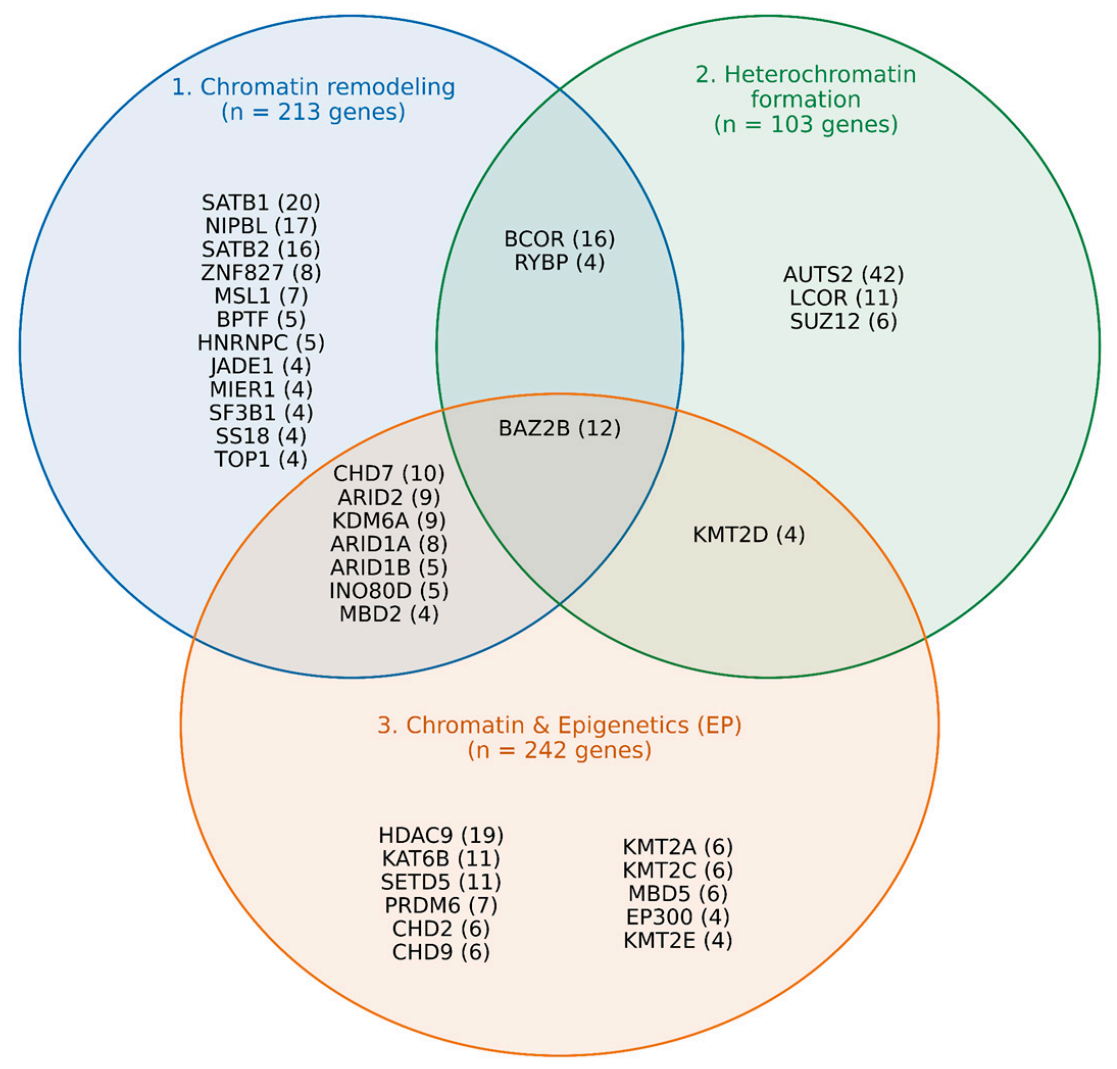
Core UCE-rich chromatin regulatory genes across three functional categories. Venn diagram showing the overlap of genes containing ≥ 4 UCEs among chromatin remodeling, heterochromatin formation, and the curated Chromatin & Epigenetics (EP) group. Numbers in parentheses following gene symbols indicate the number of UCEs within each gene. Category sizes indicate the total number of genes in each group; only genes containing ≥ 4 UCEs are displayed. The core set comprises 37 unique genes. BAZ2B is the only gene shared by all three groups.

The Chromatin & Epigenetics group similarly contained UCE-rich genes representing several regulatory mechanisms, including chromatin remodeling (*CHD2*, *CHD7*, *CHD9*, *ARID1A*, *ARID2*, *INO80D*), histone modification (*HDAC9*, *KDM6A*, *KAT6B*, *KMT2A*, *KMT2C*), and chromatin recognition/scaffolding (*BAZ2B*, *MBD5*). *BAZ2B* was particularly notable because it contained 12 UCEs and belonged to all three analyzed groups. The relationships among the UCE-rich genes in these groups are summarized in Figure 2.

Overall, UCE accumulation within chromatin-related genes was highly non-uniform, with preferential representation among multifunctional regulatory proteins rather than constitutive chromatin-maintenance machinery.

#### 2.2.2. UCEs Within RNA-Associated Genes

**Figure 3:**
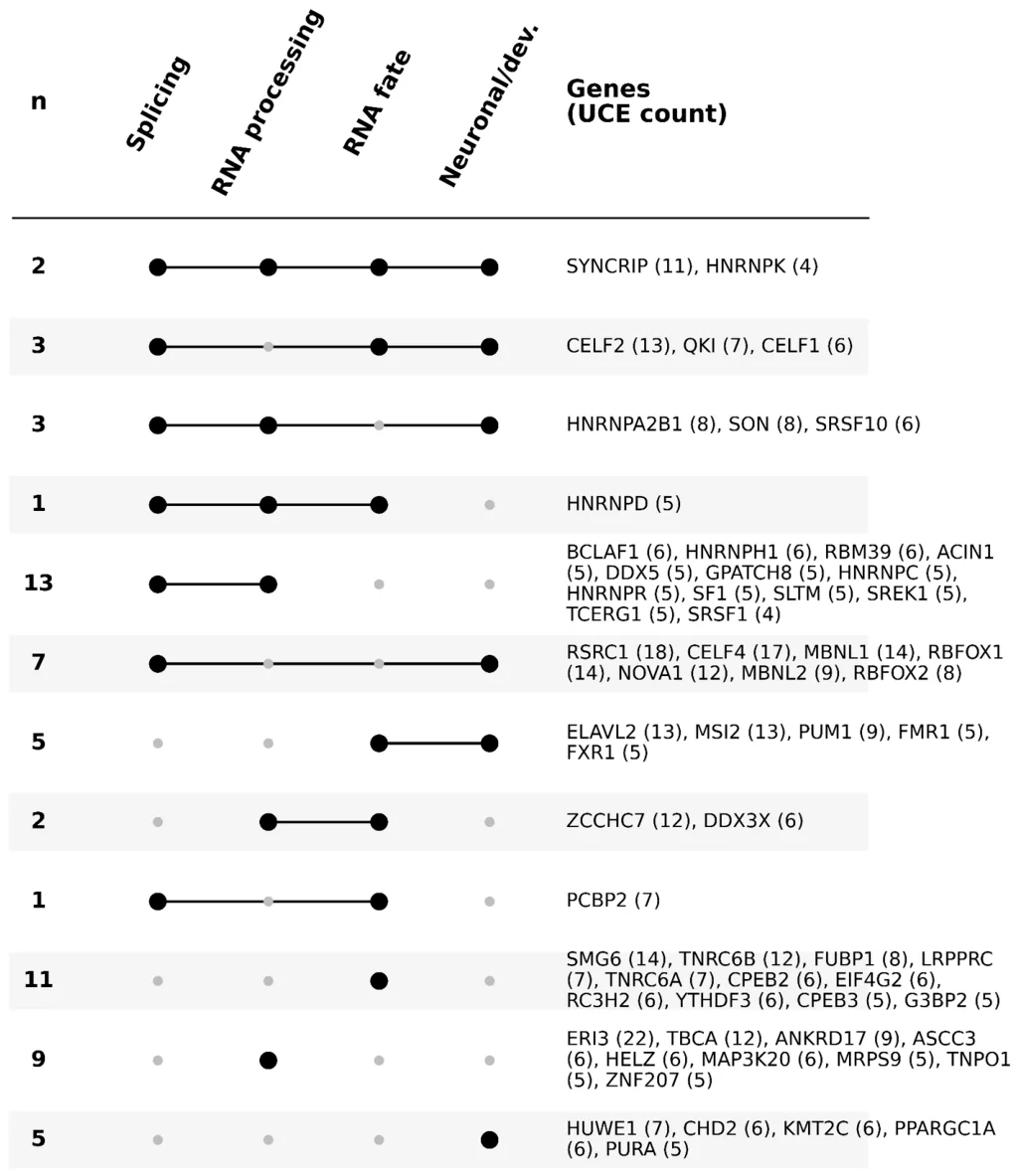
Core UCE-rich RNA-associated gene groups and intersections. The UpSet-style intersection plot shows the functional distribution of selected UCE-rich RNA-associated genes among four regulatory classes: alternative splicing/splice-site regulation, general RNA processing/hnRNP machinery, mRNA stability/localization/translation/surveillance, and neuronal/developmental RNA regulation. Each row represents an exact combination of memberships across the four functional classes. Filled circles indicate membership in the corresponding functional class, with connected circles representing genes assigned to multiple classes. The connecting lines indicate shared category membership rather than interactions between genes. Numbers in the left column indicate the number of genes in each intersection, and numbers in parentheses following gene symbols indicate the number of UCEs within each gene.

We analyzed three partially overlapping Gene Ontology categories related to RNA regulation: mRNA splicing via spliceosome (203 genes), mRNA binding (236 genes), and RNA binding (1248 genes). After removal of duplicate genes, the combined dataset contained 1377 unique genes, of which 330 (24.0%) contained at least one UCE, comprising a total of 995 UCEs. To characterize genes most strongly associated with UCEs, we selected a compact subset of UCE-rich RNA-regulatory genes for functional classification and intersection analysis (Figure 3).

UCE enrichment was concentrated in genes regulating transcript identity and fate rather than being a general feature of RNA-binding proteins. Prominent UCE-rich alternative-splicing regulators included *RBFOX1/2*, *MBNL1/2*, *CELF2*, *NOVA1*, *QKI*, *HNRNPA2B1*, *SRSF10*, and *RBM39*. A second group comprised regulators of mRNA stability, localization, and translation, including *PUM1*, *ELAVL2*, *SYNCRIP*, *PCBP2*, and CPEB-family proteins. UCE-rich genes were also represented among components of RNA surveillance and decay; for example, *SMG6* contains 14 UCEs and functions in nonsense-mediated mRNA decay, whereas *TNRC6A/B* participates in miRNA-mediated gene silencing.

Thus, UCE association extends across multiple levels of post-transcriptional regulation, with a notable representation of neuronal and developmental RNA regulators (Figure 3).

#### 2.2.3. Genes Encoding Ubiquitin Pathway Proteins

**Figure 4:**
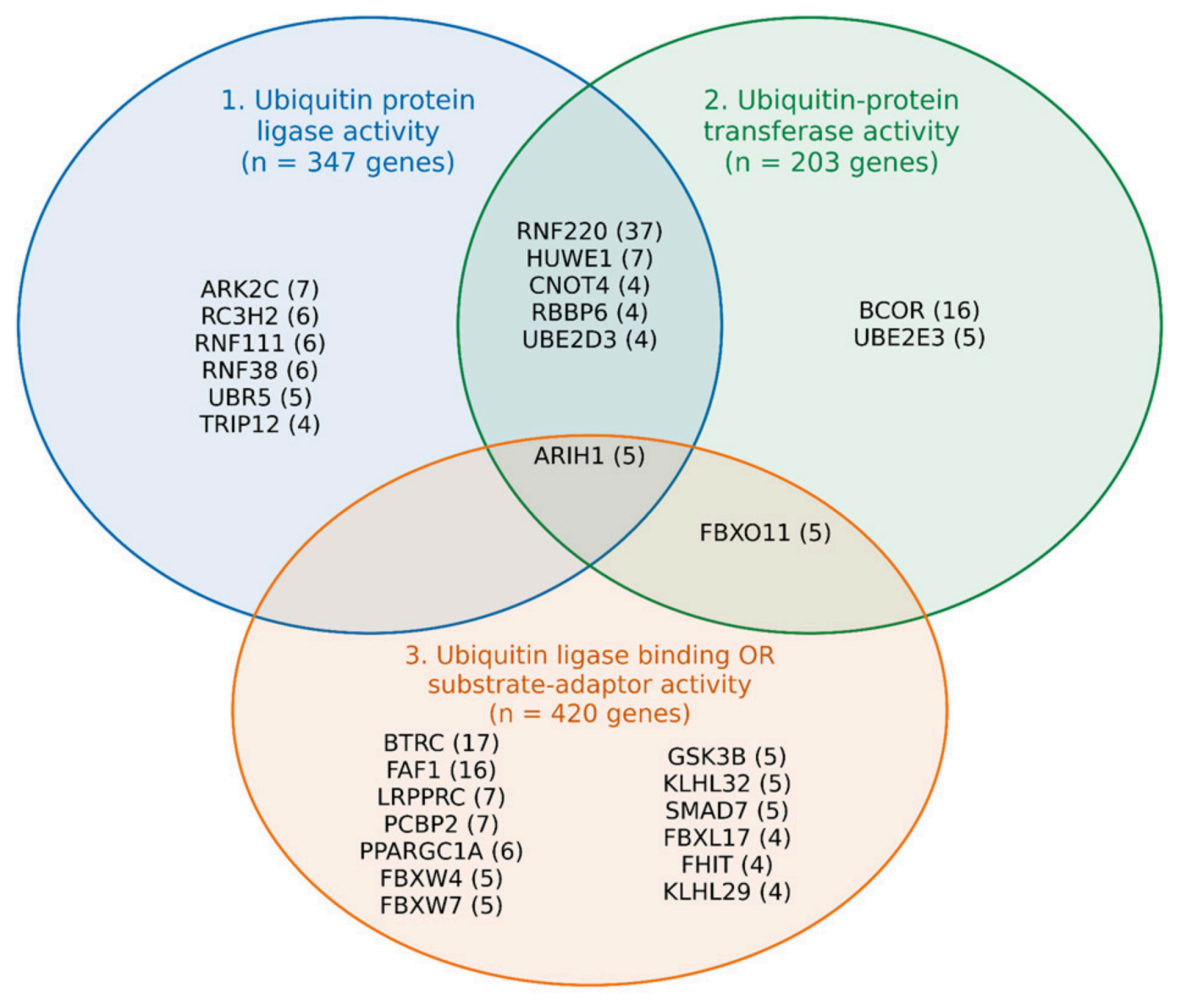
Core UCE-rich genes in ubiquitin-related functional categories. Venn diagram showing the overlap of genes containing four or more ultraconserved elements among three ubiquitin-related functional groups: (1) ubiquitin ligase activity; (2) ubiquitin-protein transferase activity; and (3) ubiquitin ligase binding/substrate adaptor activity. Numbers in parentheses following gene symbols indicate the number of UCEs within each gene. In total, 28 unique genes containing ≥4 UCEs were identified across the three functional groups. *ARIH1* was the only gene represented in all three groups. Five genes (*RNF220*, *HUWE1*, *CNOT4*, *RBBP6*, and *UBE2D3*) were shared between ubiquitin ligase and ubiquitin-protein transferase activities, whereas *FBXO11* was shared between ubiquitin-protein transferase and ubiquitin ligase binding/substrate adaptor activities.

Table 2, Table 3 and Table 4 demonstrate that ubiquitin-related genes approximately two times enriched with UCEs above random expectation. Analysis of the ubiquitin–proteasome system revealed a non-uniform association with UCEs. Among 498 genes assigned to this functional group, spanning 27.35 Mb in total, 210 UCEs were identified; 77 genes contained at least one UCE and 28 contained four or more. The enrichment was particularly pronounced in genes annotated with ubiquitin-like ligase/substrate-adaptor activity and ubiquitin-protein transferase activity, whereas the broader ubiquitin-ligase-binding category showed little enrichment. The UCE distribution within the ubiquitin system was highly uneven and concentrated in a relatively small group of genes. Particularly UCE-rich examples included *RNF220* (37 UCEs), *PSMD14* (11), *KLHL14* (8), *HUWE1* (7), *RNF38* and *RNF111* (6 each), and *UBE2E3*, *FBXO11*, *FBXW4*, *FBXW7*, *KLHL32*, and *USP34* (5 each). Several of these genes remained exceptionally UCE-rich after normalization for gene length; for example, *RNF220* contained approximately 150 UCEs/Mb, *PSMD14* 106 UCEs/Mb, and *PSMB7* 96 UCEs/Mb, compared with 6.08 UCEs/Mb for the complete protein-coding gene set. The distribution of core UCE-rich ubiquitin-related genes among functional subgroups is shown in Figure 4. The ubiquitin system is not simply a protein-degradation pathway [11].

Ubiquitination is a major regulatory mechanism controlling the abundance, localization, activity, and interactions of regulatory proteins, including transcription factors and chromatin- and RNA-associated proteins. The preferential association of UCEs with substrate-recognition, ubiquitin-transfer, and other regulatory components of this system therefore reinforces our broader observation that UCEs preferentially occur in genes involved in cellular regulation rather than routine structural or metabolic functions.

### 2.3. Expression Specifics of UCE-Rich Genes

#### 2.3.1. Adult Tissue Expression

The analysis started with a dataset of 20,065 human protein-coding genes. Among these, 2644 genes contained at least one UCE, whereas 17,421 contained no UCEs. A total of 398 genes contained ≥5 UCEs. Four exceptionally long genes (*LRP1B*, *DLG2*, *CTNNA2*, and *MAGI2*), whose high absolute UCE counts were not exceptional after accounting for gene length, were excluded. Thus, the final UCE-rich group consisted of 394 genes. As a control group, 394 genes were selected from the 17,421 zero-UCE genes by matching their gene-length distribution to that of the UCE-rich group (Supplementary Table S3).

When these genes were mapped to the GTEx (Genotype-Tissue Expression project) V11 expression dataset, 22 matched pairs had at least one member that could not be mapped. Therefore, expression analyses were performed using 372 complete UCE-rich/control pairs. We compared their expression across 68 GTEx tissues using log_2_(TPM + 1)-transformed expression values. Because GTEx consists predominantly of adult tissues, this analysis primarily characterized expression patterns in mature human tissues. The primary statistical comparison was a paired two-sided Wilcoxon signed-rank test, followed by Benjamini–Hochberg false-discovery rate (BH-FDR) correction across the 68 tissues. The results are present in Supplementary Table S4 and summarized in Table 5.

UCE-rich genes showed significantly higher and broader expression than length-matched zero-UCE control genes. Across all 68 tissues, the median mean expression was 2.663 log_2_(TPM + 1) for UCE-rich genes compared with 2.128 for controls (*p* = 3.1 × 10^−6^), with 61.6% of matched pairs showing higher expression of the UCE-rich gene. Similar differences were observed when brain and non-brain tissues were analyzed separately (Table 5). UCE-rich genes also showed significantly broader tissue expression, as indicated by a lower tissue-specificity index τ (0.777 versus 0.851; *p* = 9.2 × 10^−9^).

After BH-FDR correction, UCE-rich genes showed significantly higher expression in 65 of the 68 GTEx tissues. Among the strongest differences were those observed in the nervous system. In the brain cerebellar hemisphere, median expression was 12.48 TPM for UCE-rich genes compared with 3.91 TPM for matched controls (median paired log_2_ difference = 0.933; FDR = 3.3 × 10^−7^). Cerebellum showed a similarly strong difference (11.30 versus 4.31 TPM; FDR = 6.4 × 10^−7^). However, the effect was not restricted to the nervous system: strong differences were also observed in the cervix, uterus, colon, artery, ovary, esophagus, and numerous other tissues (see Supplementary Table S4).

Threshold-based analysis further supported the broader expression of UCE-rich genes. A typical UCE-rich gene was expressed at ≥1 TPM in 65 of the 68 GTEx tissues, compared with 58.5 tissues for its length-matched zero-UCE counterpart. At the more stringent threshold of ≥5 TPM, the corresponding values were 35.5 and 22 tissues, respectively. Thus, the broader expression of UCE-rich genes was evident independently of the τ statistic. Broader expression across adult tissues is consistent with roles beyond development. As described in Section 2.3.2, higher prenatal expression relative to matched controls indicates a developmental expression association alongside broad adult expression, rather than evidence of exclusively developmental functions.

We next examined the relationship between UCE enrichment and expression across all 2548 UCE-containing genes represented in the expression analysis. Length-adjusted UCE enrichment residuals were used to account for differences in gene length, followed by Spearman rank correlation analysis. Higher length-adjusted UCE density was significantly associated with higher overall expression (Spearman ρ = +0.220, *p* = 2.6 × 10^−29^). Higher UCE density was also associated with broader tissue expression, as indicated by a negative correlation with the tissue-specificity index τ (ρ = −0.091, *p* = 3.8 × 10^−6^), where lower τ values correspond to broader expression across tissues. Here, ρ represents the Spearman rank correlation coefficient.

#### 2.3.2. Developmental Expression

We further investigated developmental expression of UCE-rich and length-matched zero-UCE genes using the human developmental transcriptome dataset E-MTAB-6814 [12], which includes seven organs: cerebrum/forebrain, cerebellum, heart, kidney, liver, ovary, and testis (see Supplementary Table S5). The dataset comprises 297 developmental samples spanning prenatal and postnatal development, with prenatal sampling beginning at approximately 4 weeks post-conception. Of the original matched UCE-rich/control gene pairs, 372 complete pairs were represented and used for this analysis. Expression was analyzed as log_2_(RPKM + 1) and presented in Table 6.

UCE-rich genes showed significantly higher prenatal expression than length-matched zero-UCE controls in all seven organs examined (Table 6). The strongest differences were observed in cerebellum and brain, followed by kidney, whereas liver showed the smallest difference. These results indicate that elevated expression of UCE-rich genes is a general feature of human prenatal development, with a particularly pronounced effect in the developing nervous system.

To investigate prenatal developmental dynamics in greater detail, we subsequently analyzed the prenatal subset of the same E-MTAB-6814 dataset. Prenatal samples were divided into three developmental windows: 4–8, 9–13, and 16–20 weeks post-conception (WPC). This more restrictive analysis included 365 complete matched gene pairs with sufficient mapping and expression information across the prenatal windows (see Supplementary Table S6).

The strongest developmental pattern was observed in the nervous system. In the brain, the median expression excess of UCE-rich genes relative to matched controls progressively decreased from +1.018 log_2_ units at 4–8 WPC to +0.906 at 9–13 WPC and +0.762 at 16–20 WPC. The difference between the early and late prenatal periods remained significant after multiple-testing correction (FDR = 0.018). An even stronger trajectory was observed in cerebellum, where the UCE-rich expression excess decreased from +1.181 to +1.005 and +0.768 log_2_ units across the three developmental windows, respectively (early versus late, FDR = 3.4 × 10^−14^). Thus, cerebellum showed both the largest UCE-associated expression excess and the strongest concentration of this effect during early prenatal development.

Kidney showed a more modest developmental change. In contrast, heart, liver, and testis showed significantly higher expression of UCE-rich genes throughout all three prenatal windows but no convincing monotonic decrease in the magnitude of the difference.

Collectively, these analyses demonstrate that UCE-rich genes are broadly and more highly expressed across human tissues and during development. Their relative expression enrichment is particularly pronounced during early prenatal development of the nervous system. In the brain, and especially in the cerebellum, the expression difference between UCE-rich genes and length-matched zero-UCE controls progressively decreases during fetal development, suggesting that the association between UCE richness and gene activity is strongest during early stages of nervous-system development.

### 2.4. Association of UCEs with Transcription Factors

We recently demonstrated that non-KRAB transcription factor (TF) genes are approximately sevenfold enriched in UCEs [10]. Among 1292 TF genes, 440 contain one or more UCEs and 852 are UCE-free. The two groups differ markedly in gene length: the median length of UCE-containing TF genes is 56.3 kb compared with 17.5 kb for UCE-free TFs (Mann–Whitney test, *p* = 1.3 × 10^−27^). However, gene length alone does not explain UCE occurrence, as several exceptionally long TF genes are UCE-free, including *SMYD3* (759 kb), *DACH2* (685 kb), *ZNF804B* (580 kb), *MYT1L* (544 kb), and *ESR1* (479 kb). Several small TF families also contain no UCE-bearing members, including *CENPB* (11/11 UCE-free), *BED-ZF* (10/10), and *IRF* (9/9), although these family-level differences were not significant after Benjamini–Hochberg (BH) correction.

For further analysis, we defined a stringent group of 98 UCE-rich TFs containing ≥5 UCEs and having a positive length-adjusted UCE residual and compared them with the 852 UCE-free TFs while controlling for gene length. The main results are summarized in Table 7, with complete data provided in Supplementary Table S7.

In contrast to the genome-wide pattern observed for UCE-rich protein-coding genes, UCE-rich TFs showed somewhat narrower adult tissue expression than UCE-free TFs (median GTEx τ = 0.846 versus 0.823, adjusted *p* = 2.8 × 10^−5^) and somewhat lower overall expression (*p* = 0.0015). Nevertheless, they showed a significantly greater relative preference for brain expression (*p* = 0.0044; FDR = 0.0083).

A stronger distinction emerged during development. The prenatal-to-postnatal expression difference was significantly greater for UCE-rich TFs across developmental organs after adjustment for gene length (*p* = 0.0209), with particularly strong effects in the brain and cerebellum and significant differences also in the kidney and testis (Table 7). During early development (4–8 weeks post-conception), UCE-rich TFs showed significantly higher expression in the brain (*p* = 0.0109) and especially the cerebellum (*p* = 3.4 × 10^−6^). Thus, UCE richness among TF genes is strongly associated with prenatal regulation, particularly in the developing nervous system.

TF-family composition further supported this developmental association. After BH correction, UCE-rich TFs were significantly enriched for Homeodomain (29 UCE-rich versus 118 UCE-free), Paired box (5 versus 3), CUT (4 versus 1), and EBF (3 versus 0) families. In contrast, bHLH TFs tended to be underrepresented among UCE-rich genes (2 versus 82; OR ≈ 0.20), although this association did not survive BH correction (FDR ≈ 0.085).

Reactome analysis provided a consistent functional picture (Table 8 and Supplementary Table S8). Compared with UCE-containing TFs, UCE-free TFs were significantly depleted in pathways related to Developmental Biology, chromatin organization, HOX activation during differentiation and hindbrain development, and ATP-dependent chromatin remodeling. Together, these results indicate that UCE enrichment among human TFs is preferentially associated with developmental and chromatin-regulatory programs, particularly HOX-related embryonic patterning, whereas UCE-free TFs are characterized primarily by the relative absence of these programs.

UCE-rich TFs are not simply long TF genes; they form a distinctive group associated with prenatal nervous-system expression, developmental TF families, HOX biology, and chromatin regulation.

## 3. Discussion

The present study extends previous observations that UCEs are distributed highly non-randomly among human genes [3,10]. A consistent pattern emerged across several complementary analyses: UCEs preferentially occur within genes involved in complex regulatory processes, particularly transcriptional regulation, chromatin organization, RNA processing, and protein degradation via ubiquitin pathway, whereas genes associated primarily with membrane, extracellular, structural, transport, and metabolic functions tend to be relatively UCE-poor. Detailed examination of individual UCE-enriched functional groups further suggests that UCE association is not determined simply by participation in a particular molecular process. Within chromatin-related genes, for example, UCEs were concentrated in multifunctional regulators involved in developmental transcriptional programs and higher-order chromatin organization, whereas many components of constitutive chromatin-maintenance machinery were UCE-free. Similarly, among RNA-associated genes, enrichment was concentrated in regulators of alternative splicing, tissue-specific RNA processing, RNA stability, localization, translation, and surveillance rather than in the basal RNA-processing machinery. Within the ubiquitin system, UCEs preferentially occurred in regulatory components involved in substrate recognition and ubiquitin transfer rather than being uniformly distributed throughout the protein-degradation machinery. Together, these observations suggest that UCE enrichment is associated particularly with genes occupying regulatory positions at multiple levels of information flow—from chromatin and transcription to RNA processing and protein regulation.

The expression analyses provide an independent dimension to this functional association. UCE-rich genes showed higher expression and substantially broader tissue expression than length-matched UCE-free controls across adult GTEx tissues. This association became particularly striking during human prenatal development. UCE-rich genes showed significantly higher prenatal expression than matched UCE-free controls in all seven organs examined, with the largest differences in the brain and cerebellum. Furthermore, the excess expression of UCE-rich genes was greatest during early prenatal nervous-system development and progressively decreased during fetal development. Thus, UCE enrichment appears to be associated not only with broad regulatory activity but also with developmental timing, with its strongest expression signature occurring during periods of intensive establishment of developmental programs. The developmental associations reported here are consistent with those of Cummins et al. [3]. Our analysis extends this context by comparing expression of UCE-rich genes with length-matched UCE-free controls, rather than measuring transcription over UCE loci. Because both studies use E-MTAB-6814, this comparison represents an additional analysis of shared data rather than independent experimental confirmation.

## 4. Materials and Methods

### 4.1. Datasets and Databases

We used the representative human protein-coding gene dataset described previously [10], comprising 20,065 genes from Ensembl (GRCh38/hg38, release 115) [13].

dedUCE, the Database of Ultraconserved Elements, Cummins et al. 2024 [3], was kindly provided by Drs. Mattick and Cummins upon request. The database contains 12,813 human ultraconserved element (UCE) sequences and consists of two files. The first file (merged_cumulative_97_50perc.fa) contains FASTA-formatted sequences with identifiers, whereas the second file (merged_cumulative_97_50perc.bed) contains the genomic coordinates of these sequences in BED format. This subset of dedUCE sequences satisfies the following criteria: (i) sequence identity ≥ 97% identity in at least 50% of species representing 19 placental mammalian orders and (ii) sequence length ≥ 100 bp. Protein-coding sequences constitute approximately 3% of all entries in the dedUCE [3]. The mean and median lengths of UCEs in this dataset were 183 bp and 147 bp, respectively. We used UCE intervals supplied in the merged_cumulative_97_50perc.bed file without additional distance-based merging. Both dedUCE datasets, including the genomic coordinates of the protein-coding genes and UCEs used in the present analyses, are available through the Supplementary Materials of Fedorova et al. (2026) [10].

Gene Ontology (GO) annotations for human genes were obtained from the UniProt GO Annotation (GAF) file HUMAN-uniprot.gaf, downloaded from the Gene Ontology website on 12 August 2026 [14,15]. GO identifiers were mapped to the corresponding GO terms and ontology domains using the GO Basic ontology (go-basic.obo; GO release 26 July 2026), downloaded on 12 August 2026. Annotations were classified into the three GO domains: Biological Process, Molecular Function, and Cellular Component. These annotations were compiled into a working dataset (human_GO_long.tsv) available in the file Supplementary Datasets&Programs. Evidence codes were retained for all annotations. Annotation records containing the NOT qualifier were excluded from positive gene-to-GO-term assignments because they represent explicit negative annotations. Genes represented exclusively by IEA (Inferred from Electronic Annotation) evidence were excluded from subsequent functional analyses. The resulting GO annotation dataset was compared with our initial set of 20,065 human protein-coding genes using a custom Perl script. Of these, 18,702 genes (93.2%) were represented in the GO annotation dataset and were used for subsequent analyses.

Gene expression across adult human tissues was analyzed using data from the Genotype-Tissue Expression (GTEx) project [16]. Gene-level expression data were obtained from GTEx Analysis V11 using the file GTEx_Analysis_2025-08-22_v11_RNASeQCv2.4.3_gene_median_tpm.gct.gz, which contains median gene expression values, expressed as transcripts per million (TPM), for individual GTEx tissues. GTEx V11 uses GENCODE release 47 gene annotations, and RNA-seq expression estimates were generated using RNASeQC v2.4.3. GTEx gene identifiers were matched to the protein-coding genes in our UCE dataset, and expression values were transformed as log_2_(TPM + 1) for subsequent analyses. Tissue breadth and tissue specificity of gene expression were evaluated across GTEx tissues, including calculation of the tissue-specificity index τ, where values approaching 0 indicate broadly expressed genes and values approaching 1 indicate highly tissue-specific expression.

Developmental expression of UCE-rich and length-matched zero-UCE genes was analyzed using the human developmental transcriptome dataset E-MTAB-6814 [12], which includes RNA-seq data from seven major organs across prenatal and postnatal development.

Reactome pathway annotations were obtained from the Reactome Pathway Knowledgebase [17] using the downloaded pathway gene-set file ReactomePathways.gmt.zip. The file contains curated Reactome pathways represented in Gene Matrix Transposed (GMT) format, with each pathway defined by its associated set of human genes. Reactome pathway gene sets were matched to the 1292 non-KRAB transcription factor genes in our dataset, comprising 440 UCE-containing and 852 UCE-free genes. For each Reactome pathway, its representation in the two groups was compared using a two-sided Fisher’s exact test based on a 2 × 2 contingency table consisting of genes assigned or not assigned to the pathway in the UCE-containing and UCE-free groups. Odds ratios were calculated with the UCE-free group as the numerator; thus, odds ratios < 1 indicate relative depletion of a pathway among UCE-free TFs and corresponding enrichment among UCE-containing TFs. *p*-values were corrected for multiple pathway testing using the Benjamini–Hochberg false discovery rate (FDR) procedure, and pathways with FDR < 0.05 were considered statistically significant.

To account for the relationship between gene length and UCE count, we fitted a negative-binomial regression model with a logarithmic link, using the natural logarithm of gene interval length as the predictor. The expected UCE count for gene *i* was calculated as

$${\hat{\mu }}_{i}=exp\left[-10.409383+0.856802ln(L_{i})\right]$$

where $L_{i}$ is the gene interval length in base pairs. The fitted intercept and coefficient for ln(gene length) were β_0_ = −10.409383 and β_1_ = 0.856802, respectively. Length-adjusted UCE enrichment was quantified using the Pearson residual,

$$r_{i}=\frac{N_{i}-{\hat{\mu }}_{i}}{\sqrt{{\hat{\mu }}_{i}+6.827553{\hat{\mu }}_{i}^{2}}},$$

Here, $N_{i}$ is the observed UCE count, ${\hat{\mu }}_{i}$ is the expected count predicted from gene length, and 6.827553 is the fitted negative-binomial dispersion parameter. Positive residuals indicate more UCEs than predicted for a gene of that length, whereas negative residuals indicate fewer. UCE-rich genes were defined as genes containing at least five UCEs with a positive length-adjusted residual. The fitted relationship between gene length and UCE count was sublinear: expected UCE count scaled with gene length to the power of 0.857. Consequently, the model predicts decreasing UCE counts per unit length as gene length increases, supporting the use of length-adjusted residuals when identifying UCE-rich genes.

### 4.2. Generation of Randomized UCE Datasets Preserving UCE Clustering

A total of 1000 randomized UCE datasets were generated using our custom Perl script RandClusterGenerator_v3.pl, provided in the Supplementary Programs&Datasets together with a README_RandClusterGenerator file containing a detailed description of the program. The randomization procedure was designed to preserve the characteristic clustering of naturally occurring UCEs. Specifically, the program uses the empirical distribution of distances between adjacent UCEs in the human genome and randomly samples from this distribution to generate genomic positions for modeled UCEs. Consequently, the randomized datasets reproduce the clustering properties of the natural UCE distribution rather than assuming uniform and independent placement of UCEs throughout the genome. In addition, the program avoids genomic gaps and modeled centromeric regions obtained from the UCSC Genome Browser hg38 Gap track [18]. The resulting 1000 randomized UCE datasets are provided in the Supplementary RESULTS file.

### 4.3. Custom Perl Programs for Counting UCEs Within Human Genes

The numbers of UCEs located within particular groups of genes were determined using two custom Perl programs: GeneGroups_realUCE5.pl, for mapping natural UCEs, and GeneGroups_randUCE5.pl, for mapping randomized UCE datasets. Both programs were executed through the master Perl script process_UCE_HumanGenes.pl. Using Perl system calls, this script first calculates the distribution of natural UCEs within the specified group of genes and then performs the same analysis for each of the 1000 randomized UCE datasets. After completion of all 1000 iterations, the program calculates the mean and standard deviation (SD) of the number of randomized UCEs within each gene group using standard Perl statistical modules. All three programs, together with a README file containing instructions for their use, are provided in Supplementary Datasets&Programs. The analyzed gene intervals extend from the beginning of the first exon to the end of the last exon of the selected Ensembl gene models and therefore include introns and exons, including coding sequences and untranslated regions. A UCE is assigned to a gene when its genomic start coordinate falls within that gene’s start–end interval on the same chromosome. Complete containment of the UCE within the gene interval is not required, and no upstream or downstream flanking window is used. When the start coordinate falls within overlapping gene intervals, the UCE is assigned to each corresponding gene. In total, 454 distinct UCEs (3.5% of the 12,813-element dataset) were assigned to more than one protein-coding gene.

### 4.4. Statistical Analysis

For analyses based on the 1000 randomized UCE datasets, the observed number of UCEs within each gene group was compared with the distribution obtained from the randomized datasets. The mean and standard deviation (SD) of the randomized UCE counts were calculated, and Z-scores were determined as the difference between the observed and mean randomized UCE counts divided by the SD of the randomized distribution. Corresponding one-tailed *p*-values were calculated to assess UCE enrichment or depletion relative to random expectation.

To account for multiple comparisons, the Benjamini–Hochberg false discovery rate procedure was applied separately to the complete sets of 28 Cellular Component, 70 Molecular Function, and 102 Biological Process categories, and to all tested functional groups. Within each family, unadjusted *p*-values were ranked in ascending order, multiplied by the number of tests divided by their rank, and made monotonically nondecreasing by taking the cumulative minimum from the largest rank to the smallest; adjusted values were capped at 1. Calculations used unrounded values before presentation rounding. BH-adjusted *p*-values < 0.05 were considered statistically significant.

Natural and randomized UCEs were mapped to the same fixed genomic intervals for each gene group using identical assignment rules. Longer gene intervals therefore provide greater opportunities to contain UCEs in both the observed and randomized datasets. The observed-to-expected enrichment ratios consequently account for the genomic extent of the gene groups under the specified randomization model.

Differences in gene expression between UCE-rich genes and length-matched zero-UCE control genes were evaluated using two-sided Mann–Whitney U tests. For developmental expression analyses, comparisons were performed separately for each organ and developmental stage, and the resulting *p*-values were corrected for multiple testing using the Benjamini–Hochberg false discovery rate (FDR) procedure [19]. Associations between length-adjusted UCE enrichment and gene-expression characteristics were assessed using Spearman rank correlation analysis, with Spearman’s rank correlation coefficient (ρ) used to quantify the direction and strength of the associations. FDR-adjusted *p*-values < 0.05 were considered statistically significant.

## 5. Conclusions

Taken together, our results suggest that UCEs are preferentially associated with genes involved in several levels of cellular regulation, particularly those involved in the control of developmental and cellular-fate programs. However, the molecular mechanisms underlying this association remain largely unknown. Given their extraordinary length and extreme evolutionary conservation, UCEs may operate through mechanisms distinct from those of conventional regulatory elements such as promoters and enhancers. Mechanisms of gene expression regulation was discussed recently in a series of special journal issues [20,21]. UCEs present a novel class of regulatory regions differing from classical transcription factor binding and attraction of Polymerase II complex [22]. Determining how these remarkable genomic elements contribute to the regulation of their host genes—and why such stringent sequence conservation has been maintained throughout vertebrate evolution—remains a fundamental challenge for understanding their biological function. One intriguing possibility, which we have proposed previously, is that the exceptional sequence conservation of UCEs reflects unique DNA conformational properties that are integral to their biological function [9,10]. This hypothesis requires experimental verification.

## Figures and Tables

**Table 1:** UCE enrichment and depletion among genes assigned to Gene Ontology Cellular Component categories. Number of Genes and Number of UCEs indicate the genes assigned to each category and the observed UCE count within their genomic intervals, respectively. Random Expectation and SD represent the mean and standard deviation of UCE counts across 1000 randomized datasets. Ratio is the observed UCE count divided by the random expectation; values > 1 indicate enrichment and values < 1 indicate depletion. Unadjusted *p*-value is the original one-tailed *p*-value calculated using the normal approximation to the randomized count distribution. BH-adjusted *p*-value accounts for multiple testing using the Benjamini–Hochberg procedure across all 28 tested Cellular Component categories. BH-adjusted *p*-values < 0.05 are considered statistically significant. Rows are ordered by the observed-to-expected ratio, from highest to lowest; this order reflects enrichment or depletion rather than statistical significance. The entire table with statistics detail is available in Supplementary Table S1.

GO Category	Gene Count	UCEs	Random Mean	SD	Ratio	Unadjusted *p*-Value	BH-Adjusted *p*-Value
chromatin	1119	2160	336.0	36.1	6.43	<1 × 10^−100^	<1 × 10^−100^
nuclear speck	373	481	112.5	20.4	4.28	3.08 × 10^−73^	2.15 × 10^−72^
nucleoplasm	3541	3810	1090.4	65.3	3.49	<1 × 10^−100^	<1 × 10^−100^
nucleus	5695	4955	1603.3	75.3	3.09	<1 × 10^−100^	<1 × 10^−100^
chromosome	410	294	117.7	19.2	2.50	2.11 × 10^−20^	8.44 × 10^−20^
nucleolus	682	395	167.5	23.3	2.36	8.04 × 10^−23^	3.75 × 10^−22^
protein-containing complex	579	384	197.8	27.2	1.94	3.81 × 10^−12^	1.18 × 10^−11^
cytoplasm	5123	2533	1702.7	78.5	1.49	1.90 × 10^−26^	1.07 × 10^−25^
cytosol	4814	2137	1587.5	76.3	1.35	2.97 × 10^−13^	1.04 × 10^−12^
perinuclear region of cytoplasm	440	201	165.9	25.7	1.21	0.086	0.120
Golgi apparatus	849	415	356.6	38.5	1.16	0.065	0.095
centrosome	474	181	164.5	23.8	1.10	0.244	0.273
lysosomal membrane	388	118	112.1	19.9	1.05	0.383	0.383
membrane	2650	869	896.6	58.5	0.97	0.319	0.343
apical plasma membrane	341	138	146.1	24.9	0.94	0.372	0.383
mitochondrion	1502	313	344.3	35.3	0.91	0.188	0.219
endoplasmic reticulum	1051	277	310.1	35.3	0.89	0.174	0.212
extracellular exosome	2054	490	572.3	44.0	0.86	0.031	0.051
endoplasmic reticulum membrane	1080	245	302.6	32.7	0.81	0.039	0.061
focal adhesion	372	106	133.9	22.1	0.79	0.103	0.132
Golgi membrane	619	157	208.2	27.4	0.75	0.031	0.051
plasma membrane	4771	1448	1924.8	93.7	0.75	1.80 × 10^−7^	5.05 × 10^−7^
mitochondrial inner membrane	457	55	75.0	15.5	0.73	0.098	0.131
extracellular region	2131	376	553.1	46.7	0.68	7.46 × 10^−5^	1.74 × 10^−4^
cell surface	544	148	237.4	32.6	0.62	0.003	0.007
endoplasmic reticulum lumen	306	58	96.9	19.0	0.60	0.020	0.038
mitochondrial matrix	434	45	95.0	18.5	0.47	0.003	0.007
extracellular matrix	422	58	150.2	24.3	0.39	7.40 × 10^−5^	1.74 × 10^−4^

**Table 2:** Examples of UCE richness of genes encoding proteins associated with different Molecular Functions according to Gene Ontology (GO) categories. The columns are the same as described in Table 1. The entire table containing 70 rows is presented in Supplementary Table S1. BH-adjusted *p*-values were calculated across all 70 tested categories in this GO domain.

GO Category	Gene Count	UCEs	Random Mean	SD	Ratio	Unadjusted *p*-Value	BH-Adjusted *p*-Value
DNA-binding transcription repressor activity, RNA polymerase II-specific	229	644	68.6	16.3	9.39	<1 × 10^−100^	<1 × 10^−100^
RNA polymerase II cis-regulatory region sequence-specific DNA binding	1052	1785	261.9	33.1	6.82	<1 × 10^−100^	<1 × 10^−100^
sequence-specific double-stranded DNA binding	554	858	125.9	21.8	6.81	<1 × 10^−100^	<1 × 10^−100^
DNA-binding transcription activator activity, RNA polymerase II-specific	342	643	94.6	19.4	6.80	<1 × 10^−100^	<1 × 10^−100^
DNA-binding transcription factor activity, RNA polymerase II-specific	1212	1898	290.9	35.2	6.52	<1 × 10^−100^	<1 × 10^−100^
sequence-specific DNA binding	230	385	60.2	15.4	6.40	4.82 × 10^−99^	3.07 × 10^−98^
DNA-binding transcription factor activity	489	910	155.6	25.4	5.85	<1 × 10^−100^	<1 × 10^−100^
transcription cis-regulatory region binding	276	557	97.1	20.0	5.74	<1 × 10^−100^	<1 × 10^−100^
DNA binding	548	880	158.2	25.1	5.56	<1 × 10^−100^	<1 × 10^−100^
DNA-binding transcription factor binding	142	287	53.8	14.5	5.33	1.69 × 10^−58^	9.07 × 10^−58^
mRNA binding	236	300	69.5	17.2	4.32	2.98 × 10^−41^	1.39 × 10^−40^
histone deacetylase binding	104	139	32.7	11.7	4.25	5.16 × 10^−20^	1.72 × 10^−19^
RNA polymerase II-specific DNA-binding transcription factor binding	168	205	51.7	13.9	3.97	1.39 × 10^−28^	5.11 × 10^−28^
chromatin binding	308	373	96.5	18.2	3.87	1.99 × 10^−52^	9.94 × 10^−52^
protein heterodimerization activity	212	289	78.4	18.6	3.69	5.07 × 10^−30^	1.97 × 10^−29^
RNA binding	1248	937	278.0	30.9	3.37	<1 × 10^−100^	2.24 × 10^−100^
protein domain specific binding	140	127	45.7	13.1	2.78	2.72 × 10^−10^	7.60 × 10^−10^
histone binding	159	112	50.3	13.2	2.23	1.48 × 10^−6^	3.82 × 10^−6^
ubiquitin-like ligase-substrate adaptor activity	164	75	35.6	10.5	2.11	8.76 × 10^−5^	2.04 × 10^−4^
ubiquitin-protein transferase activity	203	137	67.3	15.4	2.04	3.01 × 10^−6^	7.51 × 10^−6^
ubiquitin protein ligase activity	347	172	104.2	18.4	1.65	1.14 × 10^−4^	2.58 × 10^−4^
protein-containing complex binding	165	94	59.2	15.3	1.59	0.011	0.024
protein binding	13,678	6537	4203.9	112.9	1.55	3.56 × 10^−95^	2.08 × 10^−94^
ubiquitin protein ligase binding	264	121	80.1	17.8	1.51	0.011	0.023
cytokine activity	161	34	22.9	9.4	1.48	0.119	0.177
small GTPase binding	208	92	92.9	19.1	0.99	0.481	0.481
calcium ion binding	341	110	138.6	24.6	0.79	0.122	0.179
magnesium ion binding	170	40	51.8	13.3	0.77	0.187	0.243
extracellular matrix structural constituent	123	11	63.3	16.7	0.17	8.69 × 10^−4^	0.002
olfactory receptor activity	397	0	11.0	5.4	0.00	0.021	0.040

**Table 3:** Examples of UCE richness of genes encoding proteins associated with different Biological Processes according to Gene Ontology (GO) categories. The columns are the same as described in Table 1. The entire table containing 102 rows is presented in Supplementary Table S2. BH-adjusted *p*-values were calculated across all 102 tested categories in this GO domain.

GO Category	Gene Count	UCEs	Random Mean	SD	Ratio	Unadjusted *p*-Value	BH-Adjusted *p*-Value
regulation of transcription by RNA polymerase II	1363	2051	354.5	37.6	5.79	<1 × 10^−100^	<1 × 10^−100^
mRNA splicing, via spliceosome	203	163	32.0	9.9	5.09	2.85 × 10^−40^	3.64 × 10^−39^
heterochromatin formation	103	139	31.8	10.6	4.37	2.41 × 10^−24^	2.24 × 10^−23^
regulation of DNA-templated transcription	496	705	166.1	24.6	4.24	<1 × 10^−100^	<1 × 10^−100^
chromatin remodeling	213	278	67.6	15.7	4.11	2.97 × 10^−41^	4.33 × 10^−40^
brain development	162	362	95.8	21.8	3.78	1.36 × 10^−34^	1.54 × 10^−33^
regulation of cell cycle	193	147	45.4	12.2	3.24	4.12 × 10^−17^	3.23 × 10^−16^
protein polyubiquitination	124	133	48.7	13.2	2.73	8.49 × 10^−11^	5.10 × 10^−10^
cell differentiation	219	194	75.6	17.3	2.57	3.85 × 10^−12^	2.46 × 10^−11^
angiogenesis	127	128	52.1	14.6	2.46	1.00 × 10^−7^	4.27 × 10^−7^
protein maturation	117	56	24.6	9.2	2.28	3.21 × 10^−4^	9.36 × 10^−4^
regulation of cell population proliferation	110	59	26.1	9.3	2.26	2.02 × 10^−4^	6.06 × 10^−4^
ubiquitin-dependent protein catabolic process	208	150	67.0	15.4	2.24	3.53 × 10^−8^	1.57 × 10^−7^
negative regulation of cell migration	134	157	72.2	17.3	2.17	4.75 × 10^−7^	1.86 × 10^−6^
DNA damage response	306	215	101.2	19.0	2.12	1.05 × 10^−9^	5.37 × 10^−9^
neuron differentiation	137	123	58.0	17.1	2.12	7.20 × 10^−5^	2.30 × 10^−4^
cellular response to lipopolysaccharide	109	42	20.2	8.5	2.08	0.005	0.014
protein ubiquitination	228	150	72.9	16.3	2.06	1.12 × 10^−6^	4.09 × 10^−6^
positive regulation of gene expression	300	210	112.2	21.9	1.87	3.99 × 10^−6^	1.40 × 10^−5^
regulation of apoptotic process	164	69	37.0	11.4	1.86	0.003	0.007
nervous system development	291	414	248.9	38.3	1.66	8.14 × 10^−6^	2.77 × 10^−5^
apoptotic process	235	98	64.8	15.7	1.51	0.017	0.038
cell migration	237	87	147.1	25.6	0.59	0.009	0.023
chemical synaptic transmission	171	65	114.2	23.5	0.57	0.018	0.039
immune response	301	30	54.6	13.4	0.55	0.033	0.068
cilium assembly	219	46	86.7	18.3	0.53	0.013	0.030
extracellular matrix organization	109	29	57.2	15.7	0.51	0.036	0.072
transmembrane transport	138	22	45.8	13.5	0.48	0.039	0.074
cell surface receptor signaling pathway	208	23	50.2	13.9	0.46	0.025	0.052
actin filament organization	124	13	47.8	14.0	0.27	0.006	0.016

**Table 4:** UCE enrichment of genes assigned to 24 distinct, mutually exclusive functional groups. The columns are the same as described in Table 1. BH-adjusted *p*-values were calculated across all tested groups. The entire table with statistics detail is available in Supplementary Table S2.

Code	Functional Group	Gene Count	UCEs	Random Mean	SD	Ratio	Unadjusted *p*-Value	BH-Adjusted *p*-Value
RB	RNA Binding & RNA Processing	277	264	56.2	13.5	4.70	7.56 × 10^−54^	9.44 × 10^−53^
EP	Chromatin & Epigenetics	242	258	64.4	15.8	4.01	5.35 × 10^−35^	4.46 × 10^−34^
TF	Transcription Factors	1772	1556	445.0	42.7	3.50	<1 × 10^−100^	<1 × 10^−100^
CC	Cell Cycle	79	43	22.9	9.4	1.87	0.016	0.037
UB	Ubiquitin–Proteasome	498	210	119.3	19.7	1.76	2.08 × 10^−6^	1.04 × 10^−5^
DR	DNA Replication	59	27	15.7	7.2	1.72	0.057	0.101
KI	Protein Kinases	263	182	127.5	22.9	1.43	0.009	0.024
TR	Ribosomal & Translation	355	77	60.4	13.9	1.28	0.116	0.170
VE	Vesicle Trafficking	270	78	74.7	16.1	1.04	0.418	0.455
PP	Protein Phosphatases	168	87	85.0	19.4	1.02	0.458	0.464
SI	Signal Transduction	240	106	104.1	20.6	1.02	0.464	0.464
DE	Development	74	20	21.9	9.3	0.91	0.418	0.455
IC	Ion Channels	247	132	149.1	26.5	0.89	0.259	0.324
SE	Secreted Proteins	139	9	12.5	6.2	0.72	0.290	0.345
GS	DNA Repair & Genome Stability	111	22	31.5	10.5	0.70	0.183	0.241
IM	Immune System	413	33	54.5	12.6	0.61	0.044	0.084
CY	Cytoskeleton	235	55	94.3	18.9	0.58	0.019	0.039
MI	Mitochondrial Proteins	148	9	16.1	6.9	0.56	0.150	0.208
TP	Membrane Transporters	469	82	152.0	23.2	0.54	0.001	0.005
MT	Metabolic Enzymes	339	50	94.8	19.9	0.53	0.012	0.031
ST	Stress Response	115	9	18.1	7.1	0.50	0.100	0.166
RC	Cell Surface Receptors	870	52	130.2	22.9	0.40	3.09 × 10^−4^	0.001
EC	Extracellular Matrix	131	24	70.8	17.5	0.34	0.004	0.012
AP	Apoptosis & Cell Death	41	1	6.6	4.6	0.15	0.110	0.170

**Table 5:** Comparison of expression characteristics of UCE-rich and length-matched zero-UCE genes. Column 4 shows the fraction of gene pairs with higher mean expression in UCE-rich gene compared to UCE-zero control.

Tissue Expression Comparisons	UCE-Rich Median	Control Median	% Pairs Rich > Control	*p* Value
Mean log_2_(TPM + 1), all 68 tissues	2.663	2.128	0.616	3.10 × 10^−6^
Mean log_2_(TPM + 1), 13 brain tissues	2.768	2.291	0.586	2.00 × 10^−4^
Mean log_2_(TPM + 1), 55 non-brain tissues	2.687	2.168	0.602	5.50 × 10^−6^
Tissue specificity tau (lower = broader)	0.777	0.851	0.355	9.20 × 10^−9^
Number tissues with TPM ≥ 1	65.000	58.500	0.530	3.90 × 10^−6^
Number tissues with TPM ≥ 5	35.500	22.000	0.567	2.90 × 10^−5^

**Table 6:** Prenatal expression of UCE-rich genes and length-matched zero-UCE control genes across seven human organs. Expression values represent mean gene expression summarized as log_2_(RPKM + 1) within the prenatal samples of the E-MTAB-6814 dataset. Median difference represents the paired expression difference between UCE-rich and matched control genes. FDR values were obtained after Benjamini–Hochberg correction.

Organ	UCE-Rich Expression	Control Expression	Median Difference	FDR-Adjusted *p*-Value
Cerebellum	3.16	2.07	1.06	1.10 × 10^−17^
Brain	2.92	1.93	0.93	8.90 × 10^−15^
Kidney	2.69	1.82	0.72	9.70 × 10^−12^
Heart	2.50	1.68	0.57	4.50 × 10^−7^
Ovary	2.34	1.71	0.56	1.70 × 10^−9^
Testis	2.31	1.65	0.55	5.40 × 10^−9^
Liver	1.72	1.26	0.27	4.80 × 10^−5^

**Table 7:** Prenatal-to-postnatal expression differences between UCE-rich and UCE-free transcription factors in human developmental tissues. UCE-rich TFs were defined as genes containing ≥5 UCEs and having a positive length-adjusted UCE residual. Values in the UCE-rich and UCE-free columns represent the median prenatal-minus-postnatal difference in log_2_(TPM + 1) expression for genes in each group. Positive values indicate higher expression during prenatal than postnatal development. The last column shows *p*-values for the difference in prenatal-to-postnatal expression between UCE-rich and UCE-free TFs after adjustment for gene length.

Property	UCE-Rich TFs	UCE-Free TFs	Length-Adjusted *p*-Value
Brain prenatal–postnatal	0.837	0.13	5.0 × 10^−5^
Cerebellum prenatal–postnatal	0.835	0.081	1.3 × 10^−5^
Kidney prenatal–postnatal	0.631	0.043	4.8 × 10^−4^
Testis prenatal–postnatal	0.434	0.051	0.0092

**Table 8:** Reactome pathways significantly differentially represented between UCE-containing and UCE-free transcription factors. The UCE-free and UCE-containing columns show the number of TF genes assigned to each Reactome pathway relative to the total number of genes in the corresponding group (852 UCE-free and 440 UCE-containing TFs, respectively). Odds ratios compare pathway representation in UCE-free TFs with that in UCE-containing TFs; therefore, an odds ratio < 1 indicates relative depletion of the pathway among UCE-free TFs and, conversely, enrichment among UCE-containing TFs. FDR *p*-values represent *p*-values corrected for multiple pathway testing using the Benjamini–Hochberg procedure.

Reactome Pathway	UCE-Free	UCE-Containing	Odds Ratio	FDR *p*-Value
Developmental Biology	132/852	113/440	0.53	0.007
Chromatin organization	23/852	35/440	0.32	0.0075
HOX activation during differentiation	8/852	20/440	0.2	0.0075
Anterior HOX activation in hindbrain development	8/852	20/440	0.2	0.0075
ATP-dependent chromatin remodelers	9/852	19/440	0.24	0.0307

## Data Availability

The original data presented in the study are openly available in the Supplementary files to this manuscript and online at https://fedorovlab.com/UCE5/ (accessed on 9 September 2026).

## Supplementary Materials

The following supporting information can be downloaded at: https://www.mdpi.com/article/10.3390/ijms27219981/s1.

## Abbreviations

The following abbreviations are used in this manuscript:

UCE	Ultraconserved Elements
WPC	weeks post-conception
GTEx	Genotype-Tissue Expression (GTEx) project
GO	Gene Ontology
TF	Transcription Factor
KRAB	Krüppel-associated box zinc-finger (protein domain)
