# Supplementary material for: Ultraconserved Elements Mark Genes Involved in Multilayered Regulation and Human Development
Source: Int J Mol Sci. 2026 Nov 7;27(21):9981. doi: 10.3390/ijms27219981 (PMC13764104; doi:10.3390/ijms27219981)
Supplement: Supplementary file 1 [file ijms-27-09981-s001.zip › Datasets&Programs.zip!/README_process_UCE_HumanGenes.pdf]

README_process_UCE_HumanGenes

This README describes the programs and supporting datasets required to analyze the distribution of natural and randomized ultraconserved elements (UCEs) among functional groups of human protein-coding genes.

PART 1. Running `process_UCE_HumanGenes.pl`

To run the master Perl program `process_UCE_HumanGenes.pl`, the following programs and supporting files are required:

1. **`GeneGroups_randUCE5.pl`** — analyzes UCE distributions within specified gene groups using randomized UCE datasets.
2. **`GeneGroups_realUCE5.pl`** — analyzes the distribution of natural UCEs within specified gene groups.
3. **`list`** — a text file containing the names of all gene groups to be analyzed.
4. **Gene-group files** — the current working directory must contain all files whose names are specified in `list`. Each file contains the names of all genes belonging to the corresponding functional group (e.g., `RNA_binding`). These files can be generated using `checkGenes_v4.pl`, as described in Part 2 below.
5. **`RESULTS` directory** — before running the program, create a directory named `RESULTS` in the current working directory. Output files generated by the analysis will be written to this directory.
6. **Natural UCE dataset** — `GeneGroups_realUCE5.pl` must have access to the dedUCE UCE dataset in BED format. In our implementation, the corresponding line is:

```
open(IN, "../../../UCE4/UCEmattickBED.txt")
```

where `UCEmattickBED.txt` contains the dedUCE UCE coordinates and is located in a different directory.

7. **Human protein-coding gene dataset** — the programs must have access to:

```
HPFC_v0.2_all_human_protein_coding_genes-GeneCatalog.tsv
```

This file contains the names and genomic coordinates of human protein-coding genes.

8. **Randomized UCE datasets** — `GeneGroups_randUCE5.pl` must have access to the 1,000 randomized UCE datasets generated by `RandClusterGenerator_v3.pl`, as

described in the accompanying README. In our implementation, these datasets are accessed using:

```
open(IN, "../../RandomClusters/RAND_UCE_1000/$ARGV[0]")
```

where `$ARGV[0]` specifies the name of the randomized UCE dataset being analyzed.

Once all required files, directories, and file paths have been configured, run the master program from the command line:

```
perl process_UCE_HumanGenes.pl
```

The program analyzes the observed distribution of natural UCEs among the specified gene groups and compares it with the distributions obtained from 1,000 randomized UCE datasets.

PART 2. Creating the `list` file and gene-group files using `checkGenes_v4.pl`

The Perl program `checkGenes_v4.pl` is used to generate the `list` file and the corresponding files containing genes assigned to individual Gene Ontology (GO) groups.

To run `checkGenes_v4.pl`, the following two datasets must be available in the current working directory:

1. **`human_GO_long.tsv`** — contains GO annotations for human genes.
2. **`HPFC_v0.2_all_human_protein_coding_genes-GeneCatalog.tsv`** — contains the names and genomic coordinates of human protein-coding genes.

Both datasets are provided in the Supplementary Materials.

Before running `checkGenes_v4.pl`, specify the GO domain to be analyzed in the program. For example, to analyze the Molecular Function domain, line 32 contains:

```
if ($type eq 'Molecular_Function')
```

The corresponding domain can be changed as required for analysis of other GO domains.

Run the program from the command line using:

```
perl checkGenes_v4.pl DIR_name
```

where `DIR_name` specifies the name of the new output directory that will contain the `list` file and all corresponding gene-group files.

For example:

```
perl checkGenes_v4.pl MOLECULAR_FUNCTIONS
```

This command creates the directory `MOLECULAR_FUNCTIONS`, containing the `list` file and the individual gene-group files required as input for `process_UCE_HumanGenes.pl`.
