# Supplementary material for: Ultraconserved Elements Mark Genes Involved in Multilayered Regulation and Human Development
Source: Int J Mol Sci. 2026 Nov 7;27(21):9981. doi: 10.3390/ijms27219981 (PMC13764104; doi:10.3390/ijms27219981)
Supplement: Supplementary file 1 [file ijms-27-09981-s001.zip › Datasets&Programs.zip!/README_RandClusterGenerator.pdf]

README_RandClusterGenerator

September 4, 2026

To generate randomized UCE distributions preserving the clustering pattern of natural UCEs using the Perl program `RandClusterGenerator_v3.pl`, the following supporting files must be present in the current working directory:

1. **`chr.txt`** — contains the sizes of human chromosomes.
2. **`DISTR_UCElength_final2`** — contains the distribution of distances between adjacent natural UCEs, expressed as the number of adjacent UCE pairs within defined nucleotide-distance bins.
3. **`GRCh38_gaps_UCE_annotated.bed`** — contains the genomic coordinates of assembly gaps and modeled centromeric regions in the GRCh38 human genome assembly. These regions are excluded from randomized UCE placement.
4. **`RandClusterUCE5.pl`** — Perl program that processes the distance distribution in `DISTR_UCElength_final2` and generates distances between randomized UCEs. The resulting positions are written to the intermediate file `RandPositions`.

These datasets and programs are provided in the Supplementary file **Datasets&Programs**.

Before running the program, create a new directory named **`RAND_UCE_1000`** within the current working directory.

Run the program from the command line using:

```
perl RandClusterGenerator_v3.pl
```

The program generates **1,000 randomized UCE datasets** in the `RAND_UCE_1000` directory, named sequentially:

`RandUCE_1`, `RandUCE_2`, ..., `RandUCE_1000`.

These randomized datasets preserve the clustering characteristics of the natural UCE distribution while excluding assembly gaps and modeled centromeric regions.
